# Understanding the role of sleep in suicide risk: qualitative interview study

**DOI:** 10.1136/bmjopen-2016-012113

**Published:** 2016-08-22

**Authors:** Donna L Littlewood, Patricia Gooding, Simon D Kyle, Daniel Pratt, Sarah Peters

**Affiliations:** 1 School of Health Sciences, University of Manchester, Manchester, UK; 2 Sleep and Circadian Neuroscience Institute, Nuffield Department of Clinical Neurosciences, University of Oxford, Oxford, UK; 3 Manchester Centre for Health Psychology, University of Manchester, Manchester, UK

**Keywords:** SLEEP MEDICINE, QUALITATIVE RESEARCH

## Abstract

**Objective:**

Sleep problems are associated with increased risk of suicide, independent of depression. This analysis explores narrative accounts of the role of sleep in relation to suicidal thoughts and behaviours.

**Design:**

Qualitative study, based on in-depth semistructured interviews which were analysed with an inductive, latent thematic analysis.

**Participants:**

A maximum variation sample of 18 people with experience of a major depressive episode, and suicidal thoughts and behaviours.

**Setting:**

Primary care, North West England.

**Results:**

Respondents emphasised the importance of sleep for recovery and management of their mental well-being. Moreover, three inter-related pathways were identified, whereby beliefs about sleep contributed to suicidal thoughts and behaviours. First, being awake during the biological night heightened risk of suicidal behaviours, as this was perceived to be an opportune time for a suicide attempt due to the decreased chances that a friend of family member would intervene during a suicide attempt. Additionally, the reduction in available support at night added to suicide risk. Second, failure to achieve good sleep was perceived to make life harder through contributing to core features of depression, such as negative thinking, attention difficulties and inactivity. Third, sleep acted as an alternative to suicide, by providing an escape from problems, including mental health problems, in waking life. However, this desire to sleep to escape was associated with excessive daytime sleeping, which subsequently may reinforce disturbed sleeping patterns.

**Conclusions:**

Sleep problems should be an important treatment target when working with suicidal clients. More broadly, night-time service provision should be considered when developing suicide prevention initiatives.

**Strengths and limitations of this study**

- This is the first qualitative, in-depth investigation of the role of sleep problems within suicidal pathways.
- A service user research group was actively involved in the development of the interview topic guide, recruitment strategy and supporting materials.
- Purposive sampling ensured participants with a range of sleeping problems were included within the study.
- Analysis was supported by a multidisciplinary team, which included researchers with differing areas of expertise and backgrounds.
- The sample was confined to people with experience of a major depressive episode, which may limit the transferability of the findings to other clinical groups.

## Introduction

Suicide is a prominent cause of preventable death, accounting for ∼800 000 deaths each year.^1^ Recent research highlights that individuals who experience sleep problems are at an elevated risk of suicidal thoughts and behaviours.^2–6^ This is particularly noteworthy given the high prevalence of sleep problems in healthy and clinical populations.^7^ ^8^ Specifically, it is estimated that up to 90% of people with depressive disorders experience sleep problems.^9^ Therefore, it is important to examine the putative mechanisms underlying the relationship between sleep problems and suicidality, which, thus far, have received only limited attention. It is plausible that depressed symptoms are a key driver in the sleep–suicide relationship, but a recent meta-analysis indicated that sleep problems are associated with suicidal thoughts and behaviours, independent of depression.^2^ However, it is noteworthy that more recent evidence investigating the association between insomnia and suicidality has produced mixed results.^10–12^ The linkage between nightmares and suicide appears somewhat clearer, with consistent reports of a direct relationship between nightmares and suicidal thoughts and behaviours, independent of psychopathology and comorbid insomnia.^11^ ^13^ ^14^ Nightmares have also been shown to mediate the relationship between insomnia and suicidal ideation.^15^ This suggests that there may be specific differences in the mechanisms which account for the nightmares–suicide relationship, in comparison to the insomnia–suicide relationship. However, it also remains possible that core mechanisms underpin more generic features of sleep problems such as sleep discontinuity, altered sleep architecture and poor sleep quality. For instance, a large longitudinal case–control cohort study found that poor sleep quality predicted an increased risk of suicide, across a 10-year period.^16^ In addition, recent research has indicated that a state of hyperarousal, which may be common to insomnia and nightmares, interacted with a person's sense of fearlessness about death to predict suicidal risk.^17^

In order to advance understanding of the relationship between sleep problems and suicide, further examination of inter-related psychological processes is warranted.^4–6^ Indeed, suicidal thoughts and behaviours are hypothesised to develop from interactions between psychological processes and sociodemographic factors.^18^ ^19^ Therefore, identifying the specific psychological processes which underpin the sleep–suicide relationship is important to the development of interventions targeted at reducing suicidal thoughts and behaviours.^18^ ^20^

Although research in this area is in its infancy, preliminary studies suggest that cognitive–emotional appraisals,^14^ ^15^ ^21^ and coping and emotional regulation strategies,^22^ may partially mediate the relationship between sleep problems and suicidal thoughts and behaviours. However, findings in this area have relied solely on cross-sectional questionnaire designs, which are unlikely to capture the complexity and variance associated with the experience of sleep problems and the pathways underlying suicidal thoughts and behaviours. In addition, work to date has examined mechanisms that were hypothesised a priori and hence, fail to allow for the identification of novel or additional underlying mechanisms and processes. Therefore, we conducted the first qualitative study in this area, with a view to investigating the perceptions of the role of sleep problems in suicidal pathways, and to identify the core processes which underpin this relationship. Owing to the inter-relations between sleep problems, suicidality and depression,^2^ ^9^ ^23^ this research was conducted with individuals who had experienced a major depressive episode(s).

## Methods

### Study sample

To be included in the study, all participants had to have experienced a major depressive episode as specified by the Diagnostic and Statistical Manual of Mental Disorders IV; had self-reported suicidal thoughts, feelings and/or behaviours within the past year; were aged 18–65 years; and were fluent in English. The Structured Clinical Interview for Diagnostic and Statistical Manual of Mental Disorders IV^24^ was administered by the first author to confirm past or current experience of a major depressive episode(s).

A maximum variation approach^25^ to sampling was taken to gain perspectives from participants with experiences of different sleeping problems (eg, delayed sleep onset, nightmares). Recruitment was conducted across North West England, UK, and promoted via a number of National Health Service Trust services and mental health charities. In addition, the study was advertised through websites and social media, such as, Gumtree, Twitter and Facebook.

### Procedure

Semistructured interviews followed a topic guide which was developed in consultation with a service user reference group, the members of which had experience of depression and suicidality. Questions largely focused on three domains: (1) sleep experiences at different times, that is, in general, when feeling well, during period(s) of depression and during times of suicidal thoughts and behaviours; (2) the perceived importance of sleep; (3) the consequences of ‘good’ and ‘bad’ sleep.

Interviews were conducted face-to-face by the first author (DLL) at the participants' preferred location of a private room at the University of Manchester, or at a local medical or community centre. All participants were given the opportunity to ask questions prior to signing the consent form. Interviews were audio recorded and then transcribed verbatim by the first author and any identifying information (eg, names and places) was removed at this stage.

A series of questionnaires were administered post interview to aid characterisation of the sample. Specifically, data were collected about sleep parameters (sleep disorders and quality), suicidal thoughts and behaviours, and inter-related psychological well-being factors, such as depression, anxiety and alcohol intake (see table 1).

**Table 1:** Means and ranges of participant clinical characteristics

Questionnaire	Score interpretation guidance	Mean, range
Beck Scale for Suicide Ideation^30^	Higher scores indicate greater levels of suicidal ideation, possible score range 0–38.	8.5, 0–24
Sleep Condition Indicator^31^	≤16 indicates possible insomnia disorder, as per diagnostic and Statistical Manual of Mental Disorders V diagnostic criteria, possible score range 0–32.	13.8, 4–32
The Pittsburgh Sleep Quality Index^32^	>5 indicates poor sleep quality, maximum score=21.	9.9, 1–17.5
Alcohol Use Disorders Identification Test-10^33^	>7 indicates possible hazardous and harmful alcohol use, maximum score=40.	6.8, 0–39
Beck Depression Inventory-II^34^	Higher scores indicate greater severity of depression, possible score range 0–63.	23.0, 0–51
State-Trait Anxiety Inventory-Trait portion^35^	Higher scores indicate greater levels of anxiety, possible score range 20–80.	55.3, 34–75
Brief Sleep Screen^36^	Assessment of symptoms present in other sleep disorders such as narcolepsy, sleep breathing disorder, periodic limb movement in sleep/ restless leg syndrome, circadian rhythm sleep disorder and parasomnia.	NA

NA, not applicable.

### Analysis

An inductive and latent thematic analysis was used to analyse the data.^26^ A cyclical approach was taken to data analysis and subsequent data collection, whereby each interview was initially coded prior to subsequent data generation. This allowed tentative codes and themes to be explored and developed further, as interviews progressed. Data analysis was guided by the process described by Braun and Clarke.^26^ First, data familiarisation was achieved by repeatedly reading the transcripts. Second, transcripts were coded, by conducting a cyclical process, coding both forwards through transcripts, and as new codes emerged returning back to earlier transcripts and recoding. All authors conducted initial coding independently for the first three interview transcripts. These initial codes were then discussed and compared within the research team, which captured the perspectives influenced by the different backgrounds of this multidisciplinary team. This triangulation of viewpoints improved the trustworthiness of the data.^27^ Third, initial themes were generated by grouping the identified codes based on their similarities and differences. Fourth, the themes were reviewed against the data extracted through the coding process. Instances where the data appeared to contradict the initial analysis were highlighted and subsequently discussed by members of the team to establish whether refinement of the wider analysis was necessary.^28^ ^29^ When new data failed to give further insight into the evolving themes, this was considered to signify thematic saturation and recruitment ceased.

## Results

### Participant characteristics

Means and ranges for clinical characteristics of the sample are provided in table 1. Although all had experienced suicidal thoughts or behaviours in the past year, 12 participants (67%) had previously made one or more suicide attempts. Most participants (n=16; 89%) had experienced multiple major depressive episodes during their lifetime, and ten participants were experiencing a current major depressive episode. At the time of the interviews, 13 participants reported current symptoms consistent with the threshold criteria for Diagnostic and Statistical Manual of Mental Disorders V Insomnia Disorder, as indicated by the Sleep Condition Indicator (SCI).^31^ Of these 13, 3 met quantitative criteria for sleep onset insomnia (SCI items 1) and 9 met quantitative criteria for sleep onset and maintenance insomnia (SCI items 1 and 2). Furthermore, current sleep quality for the majority of the sample was poor, based on scores from the Pittsburgh Sleep Quality Inventory^32^ (n=15; 83%). In addition, eight participants reported difficulty sleeping due to bad dreams at least once per week in the month prior to interview. Sociodemographic information about the participants is presented in table 2.

**Table 2:** Overview of participant sociodemographic characteristics

Characteristic, n=18	
Gender, n	
Male	10
Female	8
Age, years	
Mean	33
Range, years	20–60
Relationship status, n	
Single	12
Divorced/separated	3
Married/cohabiting	3
Ethnicity, n	
White British	16
Mixed	1
Chinese	1
Employment status, n	
Full time	3
Part time	7
Volunteering	3
Unemployed	3
Sick/disability	2

### Thematic analysis of beliefs about the sleep–suicide relationship

Participants placed high importance on sleep, reporting that poor sleep had detrimental consequences on their waking lives. For some, problematic sleep directly contributed to previous suicide attempts.

What's no coincidence that the times that I did jump off, or cut myself, or run in front of cars or went to the train station, one of the massive reasons of that was that I hadn't been sleeping. Just total lack of sleep. Majorly. It fucks you up. (ID1, male)

**Figure 1:**
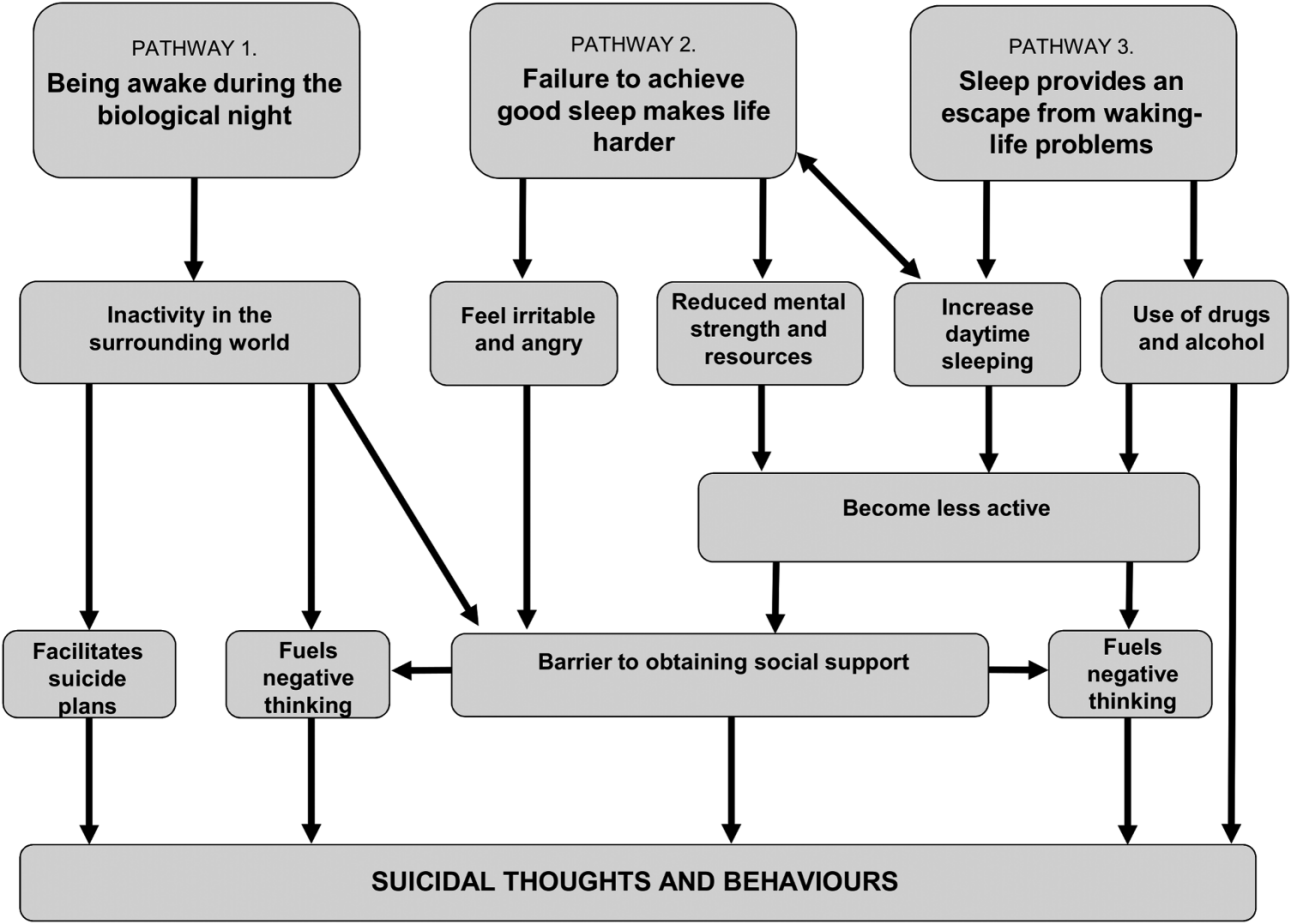
Conceptual model of beliefs about sleep and suicidal thoughts and behaviours.

Generally, participants described more indirect and complex inter-relationships between sleep and suicidal thoughts and behaviours. Three distinct, but inter-related pathways were identified whereby a belief about sleep contributed to suicidal thoughts and behaviours. These pathways were being awake during the biological night; failure to achieve ‘good’ sleep makes life harder; sleep provides an escape from waking life. An overview of a conceptual model depicting these three pathways is provided in figure 1.

- 1. Being awake during the biological night

For participants who experienced difficulty in getting to sleep or in maintaining sleep, being awake during the biological night was associated with greater vulnerability for suicidal thoughts and behaviours. Central to this was the perception of inactivity in the surrounding world. When participants were awake during the night, they noticed that the world around them was quiet and sleeping, with no visible signs of activity. Consequently, participants found it harder to effectively redirect themselves from their negative thoughts. In this sense, night-time acted as a barrier to obtaining social support and to engaging in distracting activities such as going for a walk. Without support or distractions, participants described how they were more likely to dwell on negative thoughts.

I just think because it's quiet. It's dark. Lonely. [5 second pause] it's just, different. I've always thought I wished it was light all the time, I wished we didn't need sleep, I wished the world carried on going 24/7, I wished it didn't stop at night you know because when the world stops, my heads still going and my worlds still going and, there's nobody there you know and you can't ring your mates up in the night and say you know I feel, I feel depressed. (ID8, female)

As a consequence of the inactivity in the surrounding world, participants reported increased feelings of isolation, which in turn perpetuated negative thinking.

It had something to do with feeling that everyone was gone at night. [Suicide] Just felt easier to do, I don't know. When I was generally feeling low, it's something I'd think about more. Guess the whole, like, there's no one here, no one cares, would come in to it and that would encourage it. (ID13, male)

Perceiving inactivity in their immediate world made the night an opportune time for a suicide attempt for some individuals. Participants explained that because other people were asleep, this would prevent them from intervening if they attempted suicide. Therefore, night-time could facilitate suicide plans and hide the consequences of the suicidal behaviours.

I think if I'm having those feelings [suicidal] it can be any time of the day or night it's just there's a slight increase of possibility at night because as I said before about, you could just take the pills and go to sleep, although I'm not going to ‘cos it doesn't work but, there is that slight possibility that at night you have the opportunity, whereas you could do that in the day even if you took the pills in the day you can't fall asleep or someone will find you or something, whereas they wouldn't find you at night, so suppose there's slightly more opportunity at night. (ID15, male)

- 2. Failure to achieve good sleep makes life harder

Participants had a strong desire to sleep well, driven by the belief that there were ‘good’ types of sleep that could provide the mental and physical resources needed to cope with everyday life. ‘Good’ sleep was described as long-lasting, uninterrupted, deep and coupled with a sense of feeling refreshed or energised on waking. However, in general, participants felt they were failing to achieve ‘good’ sleep. Instead, they reported dissatisfaction with their sleep, feeling they slept too little at night, or too much during the daytime, or experienced nightmares, or felt groggy as a consequence of medicated sleep. This failure to achieve ‘good’ kinds of sleep made life harder in numerous ways. First, failing to sleep well at night was associated with increased feelings of frustration, irritability and anger. Participants reported that they struggled to contain their anger, snapping at others and making aggressive outbursts. Consequently, these feelings and associated emotional exchanges acted as a barrier to obtaining to social support.

Cos when I've had like insomnia where I've like, not slept at all, like literally not slept all night, and I've been up all night long, and the next day, I'm all right for a period of time but then like in the afternoon it just hits me, and I just feel grumpy, moody, don't talk to me I'm tired, and I'll snap at people and I just think I'm so tired and then like in the evening, I feel like when I speak to anybody I'm just no company because I'm just so tired. (ID8, female)

In this sense, sleep problems or anything else which compromised access to social support was seen as problematic, given that participants felt that the absence of understanding and supportive relationships increased risk of suicidal thoughts and behaviours.

So in both, all three times its happened [has felt suicidal] is because someone or some people in my life decided not to be there any more if you get me, left me, threw me away, cast me aside, so that's what leads to the suicidal thing, as well as all the other stuff you know like with the natural depression or with the nightmares or whatever it is. (ID18, male)

Second, a further perceived consequence of not getting enough sleep at night was a reduction in mental strength and cognitive resources. This made it harder to control thoughts, focus and process information. Subsequently, participants became less active, which then fed into negative thoughts and feelings.

It's really difficult sort of keeping yourself occupied, keep yourself focused and that doesn't help coupled with the sort of lacking resources mentally, lacking the energy and again all the slump in posture, so yeah it's very difficult after a poor night's sleep to stay positive. (ID4, male)

Third, sleeping too much or too little was perceived to negatively impact activity levels. Participants who slept a lot during the day had little time for activities. This, then, fuelled negative thoughts about self-worth because the lack of activity was viewed as signifying laziness and a waste of life. Moreover, those who slept too little at night had reduced energy to partake in any activities. As a result of this, participants were less likely to engage in activities which help to protect against suicidal thoughts, such as socialising with friends. In this sense, some participants perceived excessive daytime sleeping as indirectly contributing to low mood.

My problem was oversleeping rather than under-sleeping erm. Yeah I think. I don't know, a balance because if you're sleeping too much then you're just sleeping your life away which is what I feel that I've done in many ways. (ID9, female)

[on why the participant believes sleeping too much is negative] in terms of my overall health and managing my housework and seeing friends and doing all the other things I could be doing instead of spending three odd hours sleeping in an afternoon. (ID12, female)

- 3. Sleep provides an escape from waking-life problems

When participants wanted to escape from problems in their waking life, going to sleep was perceived to be one way to achieve this. In this sense, sleep provided short-term respite from low mood or negative thoughts. Getting a break from their waking life was fundamental to the ways in which participants coped with their mental well-being. Here, sleep stopped the momentum of negative thoughts or prevented a further escalation of low mood.

It feels like a blessed release that you're unconscious for, how many hours and that you're no longer thinking about your worthlessness and that you don't want to exist. (ID11, male)

For some participants, sleep was identified as an alternative to suicide, providing an escape, albeit temporary.

I feel depressed I feel like. You'll probably think it sounds mad but, when I wanna die and I have the reasons to live if you know what I mean and I have to keep living for the purpose of my nanna and for looking after her and everything else what I'm doing. Sleep is the next best thing to being dead. So it's almost like, being asleep is like being dead and then when you wake up it's like reality hits you, and you wake up and it's like oh I need to go back to sleep just to get away from it all. (ID8, female)

And you know it's the closest thing you can get to death without actually dying. (ID17, male)

However, it is possible that the ‘escape of sleep’ may seem less attractive to those who fear sleep as a consequence of experiencing distressing nightmares. One participant spoke of the conflict between the desire to escape from her thoughts, but the reluctance of going to sleep due to the fear of experiencing more distressing nightmares.

[On why sleep is important to her] it takes me away from my thoughts, but I get a lot of nightmares so sometimes I don't wanna sleep like, I have a fear of falling asleep. Like I lie there and I think urgh, especially if I've had a nightmare the night before and its played on my mind all day, then I'll be like I don't want to sleep. (ID8, female)

The impact that sleep had on participants' subsequent waking state differed. Some viewed sleep as having a ‘resetting’ function, enabling them to return to waking-life with an improved ability to manage their moods and thoughts.

It's kind of like the anxiety rises all throughout the day, then after a good 8 or 9 hours sleep it goes right back down again, so that through the next day if it goes up its not reaching it- going really high. (ID2, female)

For others, they preferred sleeping to their waking lives, and consequently, dreaded waking up. One participant viewed sleep as a safer environment than being awake, allowing him to experience emotions during dreaming without any repercussions, unlike the reality of the waking world.

It means I'm not here erm its means I'm kind of err, I'm in control, but I'm still, I can still feel these different emotions, you know, fear, err, ecstasy, you know, kind of everything in between, erm but you're safe the whole time. (ID17, male)

Although sleeping was generally viewed as an effective way to escape problems in waking life, sleeping during the daytime was viewed negatively by some participants. This triggered a pathway, in which sleeping during the day was regarded as a barrier to activity. Participants explained that this type of daytime sleeping may contribute to disturbed night-time sleeping patterns. Here, participants described a vicious circle whereby increased daytime sleeping impaired ability to sleep at night.

[On sleeping in afternoon] I can see that it is something that I'll be tempted to do but I've got to keep it in my mind now that it's perhaps not going to do me any favours. It's a short term crutch. It's not doing me any long term favours. (ID12, female)

The use of drugs and alcohol was related to the desire to sleep and to escape problems in participants' waking life. However, perceptions regarding the effects of alcohol and drug use on sleep and mental health varied. For some, the effects were viewed as positive, bringing relaxation, aiding sleep and facilitating their ability to cope and manage their emotions and mental health.

I chose me a natural relaxation [cannabis] and pain relief it gives me, and the mental pain relief, people don't understand but that's what it gives me and that allows me to sleep and get, and be able to regularly sleep you know. (ID18, male)

However, others viewed alcohol and drug use negatively, and as contributing to psychological and physical pain, nightmares and worsening of mood. One participant commented that drinking alcohol with their medication increased risk of death, although they perceived this to be a potentially advantageous consequence.

I've used it [alcohol] to like help me fall asleep, you know like, oh I'll drink some wine or drink some vodka and you know it'll like knock me out or I'll take it with a sleeping tablet and then I've been advised, you shouldn't be doing that with that medication, you shouldn't be drinking with your medication it's dangerous, and you shouldn't mix and I'm just thinking oh I don't care cos you know just, I don't know, you just, stop caring, and I don't know you just, don't care as long as you're sleeping and, if you die it's a bonus [laughs] that's the way I was seeing it. (ID8, female)

Furthermore, some participants explained that the decision to overdose with drugs or/and alcohol was driven by an underlying preference to die in bed, during sleep, because this form of death was associated with a naturally occurring death. Dying during sleep was also perceived to be a less physically painful method of death than other alternatives.

I think because my brain accepts if I go to bed and just drifted off and died its more. It's not going to come out right this but its more right to do it that way. (ID7, male)

I suppose it must have been the idea that you'd drift off into sleep, I guess. I'm not one of those persons that could err, I'm not very good with physical pain so I couldn't slit my wrists in the bath, I couldn't do that, so the idea of just drifting off to sleep would've been quite a welcome thing, yeah. (ID15, male)

## Discussion

### Principal findings

This study was the first to use a qualitative design to explore the role of sleep in relation to suicidal thoughts and behaviours. A conceptual model was developed to illustrate the ways in which sleep problems were perceived to contribute to suicidal thoughts and behaviours via three distinct, but inter-related, pathways. These were as follows: being awake during the biological night; failure to achieve ‘good’ sleep makes life harder; and sleep provides an escape from problems in waking life.

An important finding was that being awake during the night, when biologically ‘unprepared’ for wakefulness, was perceived to increase suicidal thoughts and behaviours. Lack of access to support during the night was central to increased suicidal behaviours in two ways. First, social support was perceived to be reduced, or unavailable, throughout the night. Second, night-time decreased the chance that a friend, or family member, would notice signs of a suicide attempt (eg, facial pallor, unresponsiveness). Indeed, this is consistent with a large literature which suggests that social isolation and lack of social support are associated with mental illnesses, and suicidal thoughts and behaviours.^37–41^ Studies examining temporal patterning of suicide across the 24-hour day consistently indicate that suicides are more frequent during daytime hours.^42–45^ However, recent work by Perlis and colleagues suggests that when adjusting for the proportion of the population awake at different times of the day, relative risk of suicide is greater during the night.^46^

Disturbed sleep appeared to contribute to the development, and maintenance, of core features of depression, such as, attention difficulties and inactivity, which subsequently perpetuated negative thinking. Additionally, being awake at night provided more time for negative thinking, with reduced opportunities to access social support or distractions. Two specific types of negative thinking dominated participants' narratives: (1) rumination and (2) negative self-appraisals, both of which were associated with suicidal thoughts and behaviours, which is in accord with the broader suicide literature.^20^ ^47^ Furthermore, this is consistent with findings from a recent cross-sectional questionnaire study in which rumination partially mediated the relationship between sleep problems and suicidal behaviour.^22^

Participants in our study indicated that sleep could act as an alternative to suicide, because it provided a temporary escape from problems in waking life. This is the first time in the literature that this function of sleep has been documented. Entrapment reflects the desire to escape coupled with the perception that escape routes are blocked,^48^ which may trigger suicidal thoughts as a means to escape.^20^ ^40^ ^41^ Our results show that sleep may be seen as an alternative way to escape perceptions of entrapment. The potential downside to this occurs when people start ‘escaping’ via sleep in the daytime, because this can disrupt night-time sleep. Subsequently, this may reinforce disturbed sleep patterns, which in turn contribute to increased suicidal thoughts and behaviours. Furthermore, data from the current study suggested that nightmares may trigger fear of sleep, which then made the ‘escape of sleep’ seem less attractive. Consequently, without the escape of ‘good sleep’, perceptions of entrapment, and suicidal thoughts and behaviours may escalate. Indeed, results from a recent cross-sectional study with individuals who had experienced trauma, indicated that the relationship between nightmares and suicidal behaviour operated indirectly via defeat, entrapment and hopelessness.^14^ Taken together, these findings lay the groundwork for future studies to investigate the extent to which the role of entrapment in explaining suicidal pathways varies as a function of the specific type of sleep problem experienced.

### Limitations

There are two limitations to this study. First, although it is considered a strength that this conceptual model was developed from a purposive, maximum variation sample, findings should be complemented with empirical investigation to determine the extent to which these pathways extend to other mental illness, different levels of depression severity and across different duration and severity of sleep problems.

Second, to minimise problems with recall, the inclusion criteria specified that participants must have had experience of suicidal thoughts or behaviours in the past year.^38^ For some participants, memories regarding sleep in the more distant past were often more generalised in comparison with recollections of suicidal experiences. Therefore, it is important for future research to focus on the experiences of those individuals currently exhibiting poor sleep and suicidal behaviours.

### Future research

Our conceptual model included four psychological processes which may underpin the relationship between sleep problems, and suicidal thoughts and behaviours. These were social isolation, negative self-appraisals, rumination and entrapment. Six testable predictions can be generated from our conceptual model, which present potentially promising avenues for further investigation. First, sleep disturbances may strengthen relationships between social isolation, and suicidal thoughts and acts. Second, insufficient sleep may be associated with increased negative self-appraisals of the ability to cope with emotions and solve problems, which in turn may increase the likelihood of suicidal thoughts and behaviours. Third, sleep problems may compromise the ability to inhibit negative thoughts, which may then increase suicidal thoughts indirectly, via rumination. Fourth, sleep may alleviate suicidal thoughts and behaviours through reducing perceptions of entrapment. Fifth, perceptions of entrapment may trigger excessive daytime sleeping and reinforce disturbed sleeping patterns, which could subsequently increase suicidal thoughts and behaviour via other psychological processes such as social isolation, negative self-appraisals and rumination. Sixth, to what extent does fear of sleep moderate the role of entrapment in the relationship between sleep problems and suicide. Furthermore, while the goal of the current study was to investigate core processes across different types of sleep problems, future empirical work is required to determine whether the proposed conceptual model extends to explain the relationship between suicide and specific types of sleep problems (eg, nightmares, insomnia, hypersomnia). Finally, while outwith the scope of this qualitative study, further research should also consider the possible role of biological mechanisms in understanding the sleep–suicide relationship.^6^ For instance, based on inter-relations between suicide, serotonin and sleep regulation it has been suggested that a reduction in sleep may lead to or potentiate underlying serotonergic dysfunction, which in turn may contribute to suicidal thoughts and behaviours via impaired decision-making and reduced cognitive control of emotion.^6^

### Clinical implications

There are four key clinical implications of our findings for those working with people experiencing sleep problems and suicidality. First, night-time service provision should be a key consideration within suicide prevention strategies, given that those who are awake in the night are at an increased risk of suicide.^46^ Second, it seems prudent to help patients evaluate and identify sources of support during the night (eg, the Samaritans in the UK, Lifeline in Australia and National Suicide Prevention Lifeline in the USA). Third, the importance of resolving sleep problems in relation to recovery was emphasised, and consequently, interventions targeting poor sleep should be included within treatment plans. Fourth, clinical techniques should be used which redress rumination, especially in the context of lack of distractions during the night.

## References

[1] World Health Organisation. Preventing suicide: a global imperative. WHO, 2014 http://www.who.int/mental_health/suicide-prevention/world_report_2014/en/

[2] Pigeon WR, Pinquart M, Conner K Meta-analysis of sleep disturbance and suicidal thoughts and behaviors. J Clin Psychiatry 2012;73:e1160–7. 10.4088/JCP.11r07586 23059158

[3] Bernert RA, Joiner TE Sleep disturbances and suicide risk: a review of the literature. Neuropsychiatr Dis Treat 2007;3:735. 19300608 10.2147/ndt.s1248 PMC2656315

[4] Winsper C, Tang NK Linkages between insomnia and suicidality: prospective associations, high-risk subgroups and possible psychological mechanisms. Int Rev Psychiatry 2014;26:189–204. 10.3109/09540261.2014.881330 24892894

[5] Woznica AA, Carney CE, Kuo JR The insomnia and suicide link: toward an enhanced understanding of this relationship. Sleep Med Rev 2015;22:37–46. 10.1016/j.smrv.2014.10.004 25454672

[6] McCall WV, Black CG The link between suicide and insomnia: theoretical mechanisms. Curr Psychiatry Rep 2013;15:389 10.1007/s11920-013-0389-9 23949486 PMC3791319

[7] Roth T, Jaeger S, Jin R Sleep problems, comorbid mental disorders, and role functioning in The National comorbidity survey replication. Biol Psychiatry 2006;60:1364–71. 10.1016/j.biopsych.2006.05.039 16952333 PMC1894656

[8] Sivertsen B, Krokstad S, Øverland S The epidemiology of insomnia: associations with physical and mental health. The HUNT-2 study. J Psychosom Res 2009;67:109–16. 10.1016/j.jpsychores.2009.05.001 19616137

[9] Tsuno N, Besset A, Ritchie K Sleep and depression. J Clin Psychiatry 2005;66:1254–69. 10.4088/JCP.v66n1008 16259539

[10] Nadorff MR, Fiske A, Sperry JA Insomnia symptoms, nightmares, and suicidal ideation in older adults. J Gerontol B Psychol Sci Soc Sci 2013;68:145–52. 10.1093/geronb/gbs061 22929392 PMC3693602

[11] Nadorff MR, Nazem S, Fiske A Insomnia symptoms, nightmares, and suicidal ideation in a college student sample. Sleep 2011;34:93–8. 21203379 10.1093/sleep/34.1.93 PMC3001802

[12] Nadorff MR, Anestis MD, Nazem S Sleep disorders and the interpersonal-psychological theory of suicide: independent pathways to suicidality? J Affect Disord 2014;152-154:505–12. 10.1016/j.jad.2013.10.011 24182416

[13] Golding S, Nadorff MR, Winer ES Unpacking Sleep and Suicide in Older Adults in a Combined Online Sample. J Clin Sleep Med 2015;11:1385–92. 10.5664/jcsm.5270 26194726 PMC4661330

[14] Littlewood DL, Gooding PA, Panagioti M Nightmares and Suicide in Posttraumatic Stress Disorder: The Mediating Role of Defeat, Entrapment, and Hopelessness. J Clin Sleep Med 2016;12:393–9. 10.5664/jcsm.5592 26564386 PMC4773636

[15] McCall WV, Batson N, Webster M Nightmares and dysfunctional beliefs about sleep mediate the effect of insomnia symptoms on suicidal ideation. J Clin Sleep Med 2013;9:135–40. 10.5664/jcsm.2408 23372466 PMC3544381

[16] Bernert RA, Turvey CL, Conwell Y Association of poor subjective sleep quality with risk for death by suicide during a 10-year period: a longitudinal, population-based study of late life. JAMA Psychiatry 2014;71:1129–37. 10.1001/jamapsychiatry.2014.1126 25133759 PMC4283786

[17] Ribeiro JD, Silva C, Joiner TE Overarousal interacts with a sense of fearlessness about death to predict suicide risk in a sample of clinical outpatients. Psychiatry Res 2014;218:106–12. 10.1016/j.psychres.2014.03.036 24780448

[18] O'Connor RC, Nock MK The psychology of suicidal behaviour. Lancet Psychiatry 2014;1:73–85. 10.1016/S2215-0366(14)70222-6 26360404

[19] Steeg S, Haigh M, Webb RT The exacerbating influence of hopelessness on other known risk factors for repeat self-harm and suicide. J Affect Disord 2016;190:522–8. 10.1016/j.jad.2015.09.050 26561943

[20] Johnson J, Gooding P, Tarrier N Suicide risk in schizophrenia: explanatory models and clinical implications, The Schematic Appraisal Model of Suicide (SAMS). Psychol Psychother 2008;81:55–77. 10.1348/147608307X244996 17919360

[21] Woosley JA, Lichstein KL, Taylor DJ Hopelessness mediates the relation between insomnia and suicidal ideation. J Clin Sleep Med 2014;10:1223–30. 10.5664/jcsm.4208 25325598 PMC4224724

[22] Weis D, Rothenberg L, Moshe L The effect of sleep problems on suicidal risk among young adults in the presence of depressive symptoms and cognitive processes. Arch Suicide Res 2015;19:321–34. 10.1080/13811118.2014.986697 25517910

[23] Tarrier N, Gooding P, Pratt D Cognitive behavioural prevention of suicide in psychosis: a treatment manual. East Sussex: Routledge, 2013.

[24] First MB, Spitzer RL, Gibbon M User's guide for the structured clinical interview for DSM-IV axis I disorders SCID-I: clinician version. Washington DC: American Psychiatric Pub, 1997.

[25] Patton MQ Qualitative evaluation and research methods.CA: SAGE Publications, Inc, 1990.

[26] Braun V, Clarke V Using thematic analysis in psychology. Qual Res Psychol 2006;3:77–101. 10.1191/1478088706qp063oa

[27] Peters S Qualitative research methods in mental health. Evid Based Ment Health 2010;13:35–40. 10.1136/ebmh.13.2.35 21856603

[28] Henwood KL, Pidgeon NF Qualitative research and psychological theorizing. Br J Psychol 1992;83(Pt 1):97–111. 10.1111/j.2044-8295.1992.tb02426.x 1559146

[29] Mays N, Pope C Qualitative research in health care. Assessing quality in qualitative research. BMJ 2000;320:50–2. 10.1136/bmj.320.7226.50 10617534 PMC1117321

[30] Beck AT, Steer RA, Ranieri WF Scale for suicide ideation: psychometric properties of a self-report version. J Clin Psychol 1988;44:499–505. 10.1002/1097-4679(198807)44:4<499::AID-JCLP2270440404>3.0.CO;2-6 3170753

[31] Espie CA, Kyle SD, Hames P The Sleep Condition Indicator: a clinical screening tool to evaluate insomnia disorder. BMJ Open 2014;4:e004183 10.1136/bmjopen-2013-004183 PMC3964344 24643168

[32] Buysse DJ, Reynolds CF, Monk TH The Pittsburgh Sleep Quality Index: a new instrument for psychiatric practice and research. Psychiatry Res 1989;28:193–213. 10.1016/0165-1781(89)90047-4 2748771

[33] Saunders JB, Aasland OG, Babor TF Development of the alcohol use disorders identification test (AUDIT): WHO collaborative project on early detection of persons with harmful alcohol consumption—II. Addiction 1993;88:791–804. 10.1111/j.1360-0443.1993.tb02093.x 8329970

[34] Beck AT, Steer RA, Brown GK BDI-II, beck depression inventory: manual. San Antonio: Psychological Corporation, 1996.

[35] Spielberger CD State-trait anxiety inventory. Wiley Online Library, 2010.

[36] Wilson SJ, Nutt DJ, Alford C British Association for Psychopharmacology consensus statement on evidence-based treatment of insomnia, parasomnias and circadian rhythm disorders. J Psychopharmacol 2010;24:1577–601. 10.1177/0269881110379307 20813762

[37] Bearman PS, Moody J Suicide and friendships among American adolescents. Am J Public Health 2004;94:89–95. 10.2105/AJPH.94.1.89 14713704 PMC1449832

[38] Owen R, Gooding P, Dempsey R A qualitative investigation into the relationships between social factors and suicidal thoughts and acts experienced by people with a bipolar disorder diagnosis. J Affect Disord 2015;176:133–40. 10.1016/j.jad.2015.02.002 25706607

[39] Van Orden KA, Witte TK, Cukrowicz KC The interpersonal theory of suicide. Psychol Rev 2010;117:575–600. 10.1037/a0018697 20438238 PMC3130348

[40] Williams JMG Cry of pain. Penguin Books, 1997.

[41] Williams JMG, Crane C, Barnhofer T Psychology and suicidal behaviour: elaborating the entrapment model. In: Hawton K, ed.. Prevention and treatment of suicidal behaviour: from science to practice. Oxford University Press, 2005:71–89.

[42] van Houwelingen CA, Beersma DG Seasonal changes in 24-h patterns of suicide rates: a study on train suicides in The Netherlands. J Affect Disord 2001;66:215–23. 10.1016/S0165-0327(00)00308-6 11578675

[43] Erazo N, Baumert J, Ladwig KH Sex-specific time patterns of suicidal acts on the German railway system. An analysis of 4003 cases. J Affect Disord 2004;83:1–9. 15546640 10.1016/j.jad.2004.04.012

[44] Altamura C, VanGastel A, Pioli R Seasonal and circadian rhythms in suicide in Cagliari, Italy. J Affect Disord 1999;53:77–85. 10.1016/S0165-0327(98)00099-8 10363669

[45] Preti A, Miotto P Diurnal variations in suicide by age and gender in Italy. J Affect Disord 2001;65:253–61. 10.1016/S0165-0327(00)00232-9 11511405

[46] Perlis ML, Grandner MA, Chakravorty S Suicide and sleep: Is it a bad thing to be awake when reason sleeps? Sleep Med Rev 2015;29:101–7. 10.1016/j.smrv.2015.10.003 26706755 PMC5070474

[47] Morrison R, O'Connor RC A systematic review of the relationship between rumination and suicidality. Suicide Life Threat Behav 2008;38:523–38. 10.1521/suli.2008.38.5.523 19014305

[48] Gilbert P, Allan S The role of defeat and entrapment (arrested flight) in depression: an exploration of an evolutionary view. Psychol Med 1998;28:585–98. 10.1017/S0033291798006710 9626715

